# Quantification of Visual Texture and Presentation of Intermediate Visual Texture by Spatial Mixing

**DOI:** 10.3390/mi13020255

**Published:** 2022-02-02

**Authors:** Yuta Yoshimizu, Hiroki Yasuga, Eiji Iwase

**Affiliations:** 1 Department of Applied Mechanics and Aerospace Engineering, Waseda University, 3-4-1 Okubo, Shinjuku-ku, Tokyo 169-8555, Japan; yoshimizu@akane.waseda.jp; 2 Faculty of Core Research, Ochanomizu University, 2-1-1 Otsuka, Bunkyo-ku, Tokyo 112-8610, Japan; yasuga.hiroki@ocha.ac.jp

**Keywords:** visual texture, intermediate visual texture, visual texture display, spatial mixing

## Abstract

We proposed a method to display an intermediate visual texture by spatial mixing. In addition to color information, the visual texture is an important element that characterizes the nature of an object’s surface. While the system to display various color information has well matured in engineering, there is no method to reproduce visual textures in ambient light. In our method, the matte and glossy surfaces are used as “primary visual textures”, and an intermediate visual texture is displayed by spatially mixing the primary visual textures. In this paper, we first quantified the visual texture of an object’s surface based on measured intensities of scattered and reflected lights. Next, based on the quantification, we evaluated spatially mixed surfaces consisting of two primary visual textures, an acrylic plate and a holed sheet of drawing paper, by changing the area proportion of the two primary visual textures. Finally, a sensory evaluation showed significant differences between each intermediate visual texture, and the results corresponded to a trend in the optical evaluation. This study illustrates that visual textures could be quantified based on the intensity of scattered and reflected light and reveals the applicability of our method to the display for intermediate visual texture.

## 1. Introduction

The purpose of this paper is to quantify visual texture and to display intermediate visual textures by spatially mixing primary visual texture surfaces. In general, a texture is a visual/tactile sensation resulting from differences in a material surface, but in this paper, “visual texture” refers to visual elements of texture such as gloss, matte, etc.

Visual texture is an important element that characterizes the nature of an object’s surface in addition to color information. While the system to display various color information has well matured in engineering, as typified in displays of electronic devices, the conventional displays are not sufficient to reproduce the nature of the object’s surface, due to the lack of tunable visual textures. Visual textures are influenced by many complex factors such as light source to illuminate, shape, roughness, or motion, and many researchers have investigated how humans distinguish visual textures [1,2,3,4,5,6,7,8,9,10,11]. Some researchers have reported that the visual texture depends on the light source and angle from which an object is observed, and, from an optical point of view, the visual texture is due to the differences in the intensity of two types of light—scattered light and specular-reflected light—at the surface of the object [12,13,14]. In the field of computer graphics, these studies have been applied to present visual textures: Scattered light intensity on an object’s surface is calculated in an illuminated condition, and visual texture is virtually reproduced based on the calculated results [15,16,17]. This concept has been extended to the actual fabrication of objects, with the use of 3D printing [18] or pigment mixing [19]. Additionally, it has been proposed that the visual texture on a real three-dimensional object be presented by projecting scattering properties on the object, possibly leading to a tunable display of the visual texture [20,21]. However, these previous tunable systems are only based on a calculation of scattering properties relative to a “virtual” light source. In other words, there has never been a method for presenting visual textures tunable from gloss to matte on the actual object’s surface in any “actual” lighting environment.

In this paper, we attempted to present visual textures in a tunable manner in an actual lighting environment, which is inspired by the halftone technique in color presentation. The halftone technique tunes color as follows: As an example of gray color reproduction, two types of primary color such as black and white are firstly prepared, and intermediate color (i.e., gray) is reproduced by arraying discrete dots of one in the continuous area of the other; the intermediate color can be tuned by changing the spatial ratio of the dots. Based on the previous reports that the visual texture changes according to the scattered light intensity [12,13,14], we supposed that visual texture can be tuned by a similar approach, that is, by mixing two surfaces that show different angular distributions of reflected light intensity. In this paper, we prepared two “primary visual textures” of glossy smooth surfaces and matte rough surfaces. First, we quantified the visual texture of an object’s surface based on the measured intensity of scattered and reflected lights. Then, we quantified surfaces mixing two primary visual textures through the same optical measurement. Finally, we carried out a sensory evaluation to investigate whether it is possible to present intermediate visual texture by our method and how the results of optical measurement corresponded with human vision.

## 2. Quantifying Visual Texture

**Figure 1:**
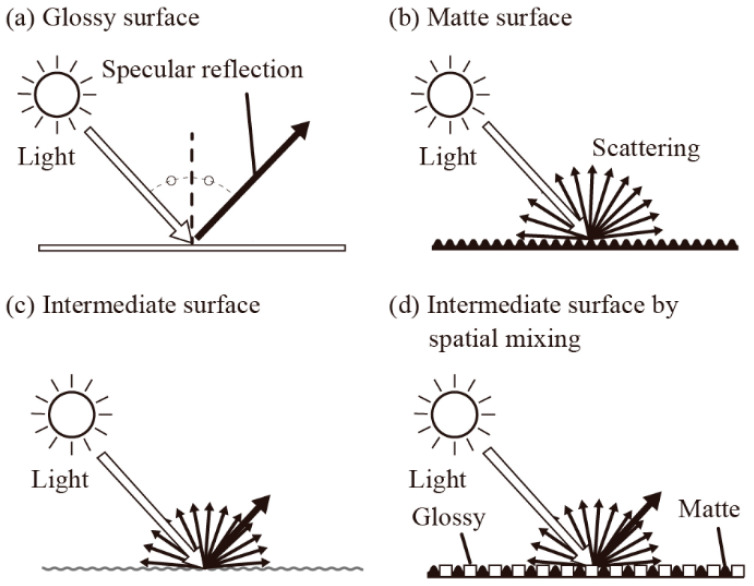
Specularly reflected or scattered light from the material surface: (**a**) glossy surface reflects the incident light; (**b**) matte (rough) surface scatters the incident light; (**c**) intermediate surface reflects and scatters the incident light; (**d**) the light distribution of the intermediate surface is produced by spatially mixing glossy and matte surfaces.

We can roughly classify light reflection into two major types—namely, specular reflection, as shown in Figure 1a, and scattering, as shown in Figure 1b. Ideally, glossy surfaces have specularly reflected light, and matte (rough) surfaces have scattered light. As the surfaces showing the visual texture between glossy and matte have both specularly reflected and scattered lighting conditions, as shown in Figure 1c, the intermediate visual textures may be characterized based on the differences between scattered and specularly reflected light intensity. Our proposal of a “spatially mixed visual texture”, as shown in Figure 1d, was considered to include both of the two types of reflection as well, and thus, we quantified visual textures using the ratio between the intensity of specularly reflected light and that of the scattered light.

**Figure 2:**
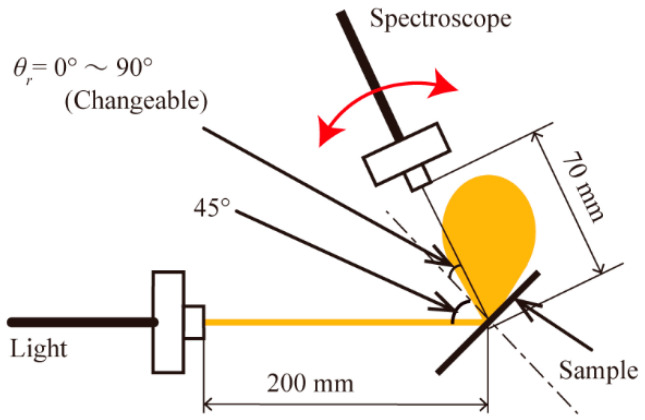
Measurement setup for the angular intensity distribution of the reflected light. The sample was fixed at 200 mm away from the tip of the optical fiber connected to the light source. The incident angle of light was fixed at 45°. The distance between the tip of the optical fiber connected to the spectroscope, and the sample was 70 mm. The core diameter of the fiber is 400 µm.

**Figure 3:**
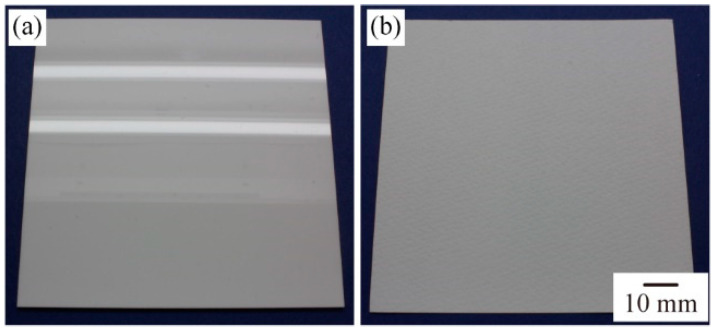
The surfaces used as primary visual textures: (**a**) the white acrylic plate with a glossy surface; (**b**) the white drawing paper with a matte surface.

We measured the angular intensity distribution of the light that was reflected on the two sample surfaces with different visual textures. The measurement setup is shown in Figure 2. The distance between the tip of the optical fiber connected to the spectroscope and the sample was set to 70 mm, to measure the intensity of the mixture of specularly reflected light and scattered light. Measurements were taken every 5° while rotating the receiving angle *θ_r_* (°) from 0° to 90°. Between the *θ_r_* of 40° to 50°, measurements were taken every 1°. We used a 100 mm × 100 mm acrylic plate (Mitsubishi Rayon Co., Ltd., Tokyo, Japan), as shown in Figure 3a, and a sheet of white drawing paper (Sunflower M drawing paper, Muse), as shown in Figure 3b, which were a sample with a glossy surface and that with a matte surface, respectively.

**Figure 4:**
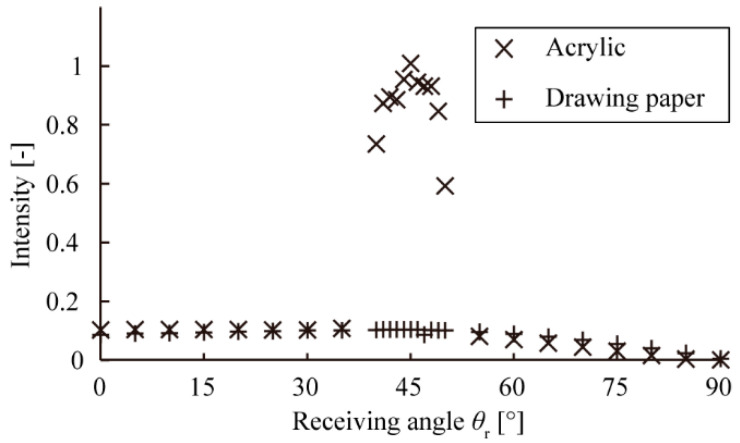
Angular intensity distributions of the reflected light from the acrylic plate and drawing paper.

Figure 4 shows the resultant angular intensity distribution of the scattered light. For the acrylic plate, there was a peak at *θ_r_* of 45°, and the distribution was almost constant at the other angles, which showed the effect of specular reflection. For the drawing paper, the distribution was almost constant at all angles, which showed the effect of scattering. Considering the distribution was almost constant between the *θ_r_* range of 0° to 40°, for both sample surfaces, and a peak appears around 45° for only the surface showing specular reflection profile, we defined a scattering index *S* (−) that was the ratio of the scattering intensity *r*_15_ (−) at *θ_r_* = 15° to the scattering intensity *r*_45_ (−) at θr = 45°. *S* can be written as follows:

(1) $$S=\frac{r_{15}}{r_{45}}$$

Ideally, objects with rough surfaces have a large *S* value, and those with glossy surfaces have a small *S* value. In Figure 4, the *S* value for the acrylic plate was 0.103 (nearly 0), and that for the drawing paper was 0.906 (nearly 1). We thus confirmed that the *S* could be used as an indicator for the optical quantification of visual texture.

## 3. Optical Evaluation of Prepared Samples by Spatially Mixing Different Surfaces

**Figure 5:**
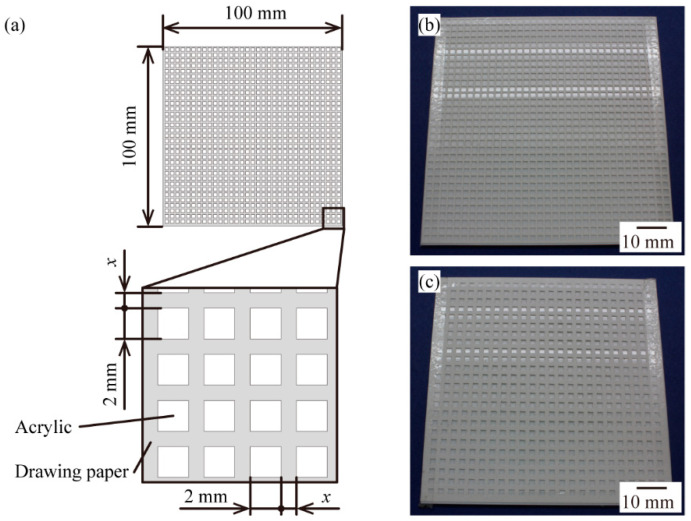
Samples for intermediate visual texture by spatial mixing: (**a**) schematic view of a sample. x (mm) denotes the length of the interval between the holes; (**b**) the sample of x = 1 mm (the proportion of acrylic plate *α* = 0.44); (**c**) the sample of x = 2 mm (*α* = 0.25).

We examined whether it is possible to tune the angular intensity distribution of the scattered light by spatially mixing two different surface materials. As shown in Figure 5a, we prepared 100 mm × 100 mm surface samples including two layers—an acrylic plate with a glossy surface at the lower layer and a piece of drawing paper with a matte surface at the upper layer. In the paper layer, 2 mm × 2 mm square holes were made in even intervals by using a cutting plotter (CE 6000-40, Graphtec, Yokohama, Japan). By changing the intervals between the square holes, the proportion of acrylic plate *α* (−) was controlled. We prepared two types of samples: the sample of *α* = 0.44, with 1 mm intervals between the holes (Figure 5b), and the sample of *α* = 0.25, with 2 mm intervals between the holes (Figure 5c).

**Figure 6:**
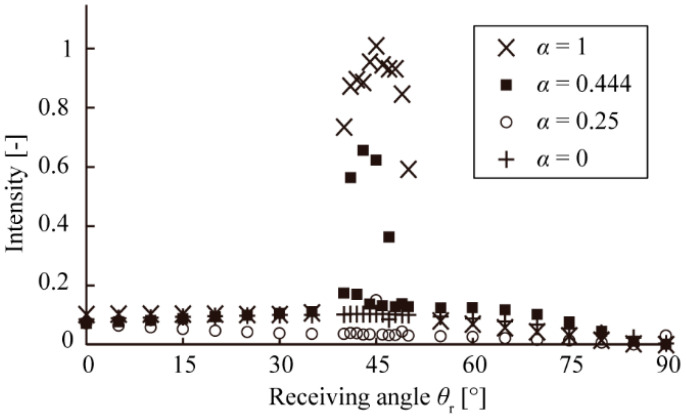
Angular intensity distributions of the reflected light from *α* = 1 (the acrylic plate), *α* = 0.44, *α* = 0.25, and *α* = 0 (the drawing paper).

**Figure 7:**
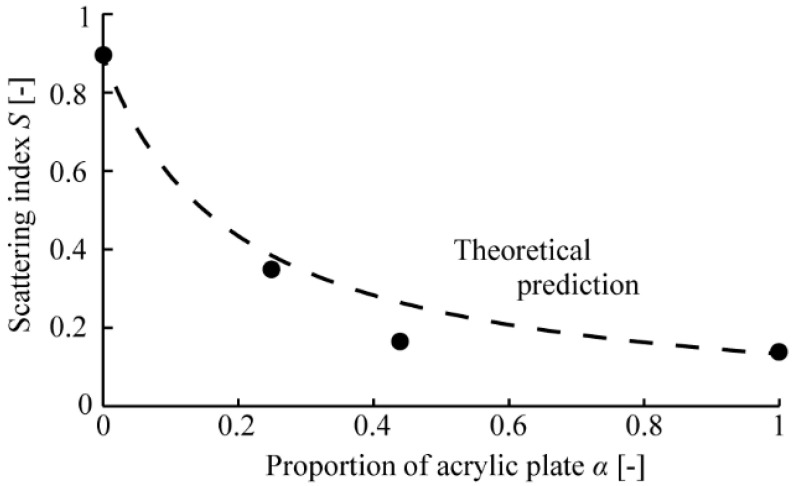
Scattering indexes of the fabricated samples.

Figure 6 shows the results of measuring the angular intensity distribution of specularly reflected and scattered lights on the surface samples. We calculated *S* from these results, and the relationship between *α* and *S* is shown in Figure 7. Provided that *α* determines the scattering intensity of a sample that juxtaposes an acrylic plate with drawing paper, the scattering intensity *r’*_15_ (*α*) (−) at *θ_r_* = 15°, and scattering intensity *r’*_45_ (*α*) (−) at *θ_r_* = 45° of a sample for each *α* (−) can be determined by the following equations:

(2) $$r_{15}^{\prime }\left(\alpha \right)=\alpha \times r_{a,15}+\left(1-\alpha \right)\times r_{d,15},$$

(3) $$r_{45}^{\prime }\left(\alpha \right)=\alpha \times r_{a,45}+\left(1-\alpha \right)\times r_{d,45},$$

where the scattering intensities at *θ_r_* on the acrylic plate and drawing paper are *r_a,θ_* and *r_d,θ_*, respectively. Therefore, based on Equations (1)–(3), the theoretical *S* for each *α* (−) is

(4) $$S\left(\alpha \right)=\frac{\alpha \times r_{a,15}+\left(1-\alpha \right)\times r_{d,15}}{\alpha \times r_{a,45}+\left(1-\alpha \right)\times r_{d,45}}$$

The broken line in Figure 7 is the theoretical prediction curve of Equation (4). Although the measured values from samples with *α* = 0.25 and 0.44 were slightly smaller than the theoretical values, the theoretical curve from Equation (4) fitted in measured values.

## 4. Sensory Evaluation

**Figure 8:**
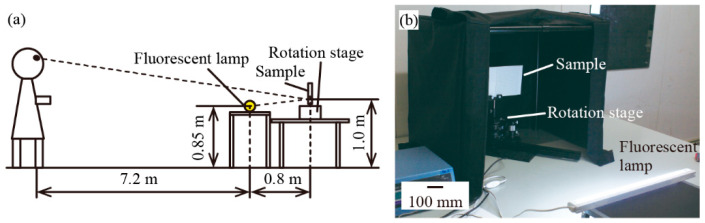
The sensory evaluation setup: (**a**) schematic view; (**b**) visual form. For the subject to be able to observe the evaluation sample at various illuminating angles, we placed the evaluation sample on top of a rotatory stage that made it possible to change the angle of the evaluation samples. The fluorescent lamp was placed 0.8 m away from the evaluation samples. The samples were evaluated in a dark room without any other light source.

We carried out a sensory evaluation to show that the intermediate visual texture could be presented by spatially mixing different surfaces and to explore the relationship between human perception and optical profiles. Humans can sense visual textures such as glossy and matte under a certain light source when they observe reflected light from a single surface, which originates from light sources with various angular conditions. In other words, as humans see an object, they sense through the visual textures that the reflected image of the light source is clearly observed on a glossy surface and not clearly observed on a matte surface. For example, when light from a white fluorescent lamp (30 W) is irradiated to a white acrylic plate having a glossy surface and white drawing paper having a matte surface, one can observe the reflected image of the fluorescent lamp on the glossy acrylic plate but observes almost no reflected image on the rough drawing paper. Based on these considerations, we carried out a sensory evaluation on visual textures by comparing the extent of the reflection of the fluorescent lamp. Figure 8 shows the setup for the sensory evaluation.

The subjects evaluated the sample, standing approximately 8 m away from it. The distance between the sample and the subjects was determined considering human eye resolution. In this paper, we used a sample with a 2 mm square juxtaposed surface. The resolution of human eyes to distinguish color is believed to be 1/60° for a human with a visual acuity of 1.0 [22], and in order to fall below the resolution capable of distinguishing a 2 mm square juxtaposed surface, it is necessary to be around 4.7 m away from the sample. Since a subject’s visual acuity varies from person to person, we set the position of the subject to be approximately 8 m away so that the experiment could allow for visual acuity of 1.0 or more. The sensory evaluation was carried out with the help of 13 male and female subjects in their 20s. The subjects were asked to simultaneously view two surface samples with different surface area proportions, and they were asked to choose one between the following three options: “The left has a greater reflection of the fluorescent lamp”, “The right has a greater reflection of the fluorescent lamp”, or “The degree of reflection does not change for either”. The evaluation sample was rotated so that the angle changed ±30° to the left and right after observing the sample from the front. This evaluation was performed for all six combinations (because two out of four surface samples were selected on each test) in total.

We analyzed the results using Scheffe’s method of paired comparisons (Nakaya Variation) [23]. Scheffe’s method is an approach utilized to compare the data originating from one’s subjectivity such as sensory evaluation. The details are as follows: Scheffe’s method of paired comparison makes up the distance scale from the score data based on paired comparisons, and it creates comparison pairs by combining more than three evaluation samples, as in this case; then, it incorporates the results of these paired comparisons into an analysis of variance after evaluating them. In this paper, since a subject was able to carry out spatial comparisons of two surface samples at the same time, there was no need to consider the sequential effects. In addition, since each subject performed a paired comparison for all combinations so that the sensory evaluation could be performed even when the number of subjects was small, we used the Nakaya Variation, in which each subject performs a paired comparison of all combinations, and the sequential effect is not taken into consideration. The results are shown on the evaluation chart for all subjects in Table 1. The evaluation value denotes the average of the following results: 1 point means that a subject chooses the correct option, −1 point means that a subject chooses the wrong option, and 0 point means that a subject could not sense the difference; these values verified whether there were significant differences in the results for the sensory evaluation.

**Figure 9:**
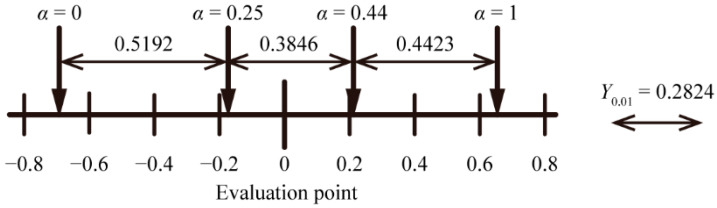
Scale diagram of evaluation points for each sample.

**Figure 10:**
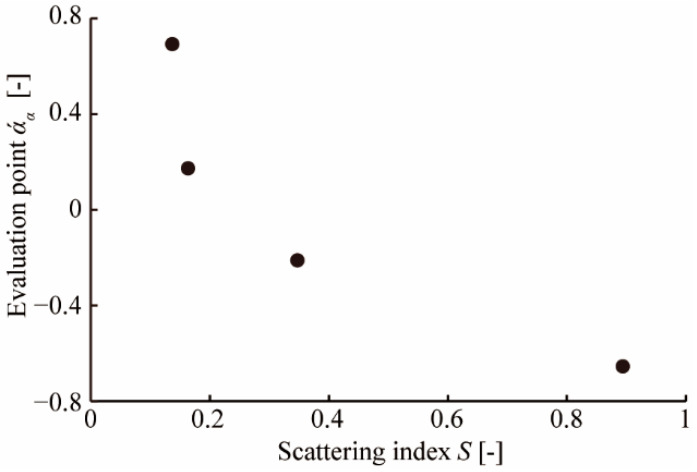
Evaluation points *άα* with scattering index *S*. The sensory evaluation was correlated with the optical measurement because *άα* is smaller as *S* is larger.

We calculated the evaluation point *άα* (-), as shown in Table 2. Figure 9 is a scale diagram, which plotted each *άα* of Table 2. We can judge there to be a significant difference when the distance between each *άα* is far greater than the yardstick (*Y*) (−) [23]. In this condition, *Y*_0.01_, denoting *Y* at a critical region of 0.01, was 0.282. The distance between each *άα* exceeded *Y*_0.01_, according to Figure 9, indicating that significant differences were visible with any surface sample. In other words, we confirmed that the spatial mixing of two different surface samples could present intermediate visual textures. When comparing the results of the optical evaluation with those of the sensory evaluation, we found that the sensitivity of the human eye to the surface of the object changes according to the scattering index of optical evaluation, as shown in Figure 10. These results indicated that visual texture through human sensation can be roughly quantified by scattering index obtained through optical measurements.

## 5. Conclusions

In this paper, we proposed a method to display an intermediate visual texture by spatial mixing. We showed that, similarly to halftone, spatially arranging two visual texture surfaces as “primary visual texture” enabled the presentation of an intermediate visual texture. We measured the angular intensity distribution of the scattered light when parallel light was irradiated on the surface of samples with different visual textures; as a result, we confirmed that the ratio between the intensity of specularly reflected and scattered lights varied on surface nature such as glossy or matte. We defined the scattering index *S*, which denoted the intensity ratio between specularly reflected light and scattered light, to quantify visual texture, and acquired S values of the acrylic plate and the drawing paper. With two samples (*α* = 0.25 and *α* = 0.44), combining an acrylic plate and a sheet of holed drawing paper, we confirmed that *S* could be changed by spatially mixing the primary visual textures. Furthermore, we confirmed that *S* was tunable, by changing the areal proportion of primary visual textures: The tunability was supported by the results that the calculated theoretical *S* fitted the trend in measured values. Finally, we carried out a sensory evaluation using Scheffe’s method of paired comparisons, with 13 subjects and following 4 samples: *α* = 0, *α* = 0.25, *α* = 0.44, and *α* = 1. Analyzing the result of sensory evaluation, significant differences were confirmed between each sample because the distance between each evaluation point *άα* exceeded *Y*_0.01_ = 0.282. This result indicates the spatial mixing of primary visual textures can present intermediate visual textures. In addition, the trend of the sensory evaluation was similar to that of *S*. Therefore, visual texture can be quantified through optical measurements to some extent and controlled by spatially mixing the primary visual textures.

## Figures and Tables

**Table 1:** Evaluation value table for all cases.

Area Proportion *α* (-)	0	0.25	0.44	1
0	-	0.615	1.00	1.00
0.25	-	-	0.462	1.00
0.44	-	-	-	0.769
1	-	-	-	-

**Table 2:** Calculation of the evaluation point *άα*.

Area Proportion *α* (-)	0	0.25	0.44	1
0	0	0.615	1.00	1.00
0.25	−0.615	0	0.462	1.00
0.44	−1.00	−0.462	0	0.769
1	−1.00	−1.00	−0.769	0
Evaluation point *άα* (average of the above values)	−0.654	−0.211	0.173	0.692

## Data Availability

Not applicable.
